# Recurrent ventricular asystole during octreotide infusion in a patient with pancreatic neuroendocrine tumor: a case report

**DOI:** 10.1186/s13256-026-05958-4

**Published:** 2026-03-16

**Authors:** Nicholaos Mansolas, Ian Nesbitt

**Affiliations:** 1 Critical Care, Freeman Hospital, Newcastle Upon Tyne, UK; 2 Anaesthesia and Critical Care, Freeman Hospital, Newcastle Upon Tyne, UK

**Keywords:** Adverse drug reaction, Bradyarrhythmia, Case report, Octreotide, Peri-operative care

## Abstract

**Background:**

Somatostatin analogs such as octreotide are frequently used in the prevention of carcinoid syndrome in the perioperative period following neuroendocrine tumor resection. This is the first reported case of ventricular asystole associated with postoperative intravenous octreotide infusion in a patient with previous congenital cardiac surgery.

**Case presentation:**

We present the case of a 62-year-old white British male patient with a history of previous congenital cardiac surgery who sustained multiple episodes of ventricular asystole and associated loss of cardiac output during octreotide infusion. Recurrent asystole ceased following discontinuation of the octreotide infusion.

**Conclusion:**

Physicians caring for patients receiving intravenous octreotide should be aware of the potential arrhythmogenic adverse effects. While specific risk factors for octreotide-associated arrhythmias are not generally understood, previous cardiac surgery could potentially be one of these. Risk stratification guidance regarding patient groups requiring heightened monitoring while receiving intravenous octreotide is required. Monitoring recommendations may involve invasive blood pressure monitoring and telemetry in these cases.

## Background

Incidence of neuroendocrine tumors has increased in recent decades [1]. Depending on location and organ involvement, neuroendocrine tumors can cause hypersecretion of serotonin and other vasoactive substances, leading to symptoms of carcinoid crisis (with signs and symptoms of bronchospasm and hemodynamic instability) and carcinoid syndrome (abdominal cramping, diarrhea, and flushing) [2, 3]. Treatment of neuroendocrine tumors can involve medical, surgical, and interventional radiological interventions [3]. The mainstay of prophylaxis and medical management of carcinoid crisis and/or syndrome associated with neuroendocrine tumors involves administration of somatostatin analogs (for example, first-generation somatostatin analogs including lanreotide and octreotide, and second-generation somatostatin analogs such as pasireotide) [3].

Somatostatin analogs are also often used perioperatively in patients receiving curative surgery for neuroendocrine tumors [4]. Intraoperative manipulation of these tumors can cause fluctuating release of vasoactive substances and serotonin. Therefore, perioperative administration of somatostatin analogs is frequently used to mitigate against hemodynamic compromise and bronchospasm in carcinoid crisis, and reduce the symptoms of carcinoid syndrome [5]. The British National Formulary documents cardiac arrhythmias as a common side effect of somatostatin analogs, and specifically, sinus bradycardia as a common side effect of octreotide [6]. No mention is made to specific patient groups in which these deleterious adverse cardiovascular effects are more likely [6]. Risk stratification and guidance on cardiac monitoring is limited in patients on octreotide infusion with preexisting cardiac pathology.

## Case presentation

In this case report, we describe a case of a white British 62-year old male patient undergoing elective robotic pancreatectomy and splenectomy for a pancreatic neuroendocrine tumor identified on computed tomography scan. The patient had a history of congenital partial anomalous pulmonary venous drainage (PAPVD; right superior vein to superior vena cava), and underwent repair with re-baffling of the right superior portal vein to left atrium at the age of 44 years. Other relevant past medical history included implantation of a loop recorder (implanted at age 58 years, explanted 2 years later), type two diabetes mellitus, hypertension, and prostate cancer treated with radical radiotherapy.

Previous 24-h Holter monitoring for investigation of palpitations revealed occasional ventricular ectopics. Preoperative echocardiography showing normal left ventricular cavity size and wall thickness, normal left ventricular systolic function (ejection fraction > 55%), a dilated right ventricle with mildly impaired right ventricular function, and mild tricuspid regurgitation.

Octreotide infusion at a rate of 50 µg per hour was commenced intraoperatively and continued postoperatively on the high-dependency unit. A central venous catheter was inserted preoperatively with X-ray confirmation of its tip confirmed in the superior vena cava. An extended electrolyte panel revealed all values were within accepted ranges.

**Fig. 1:**
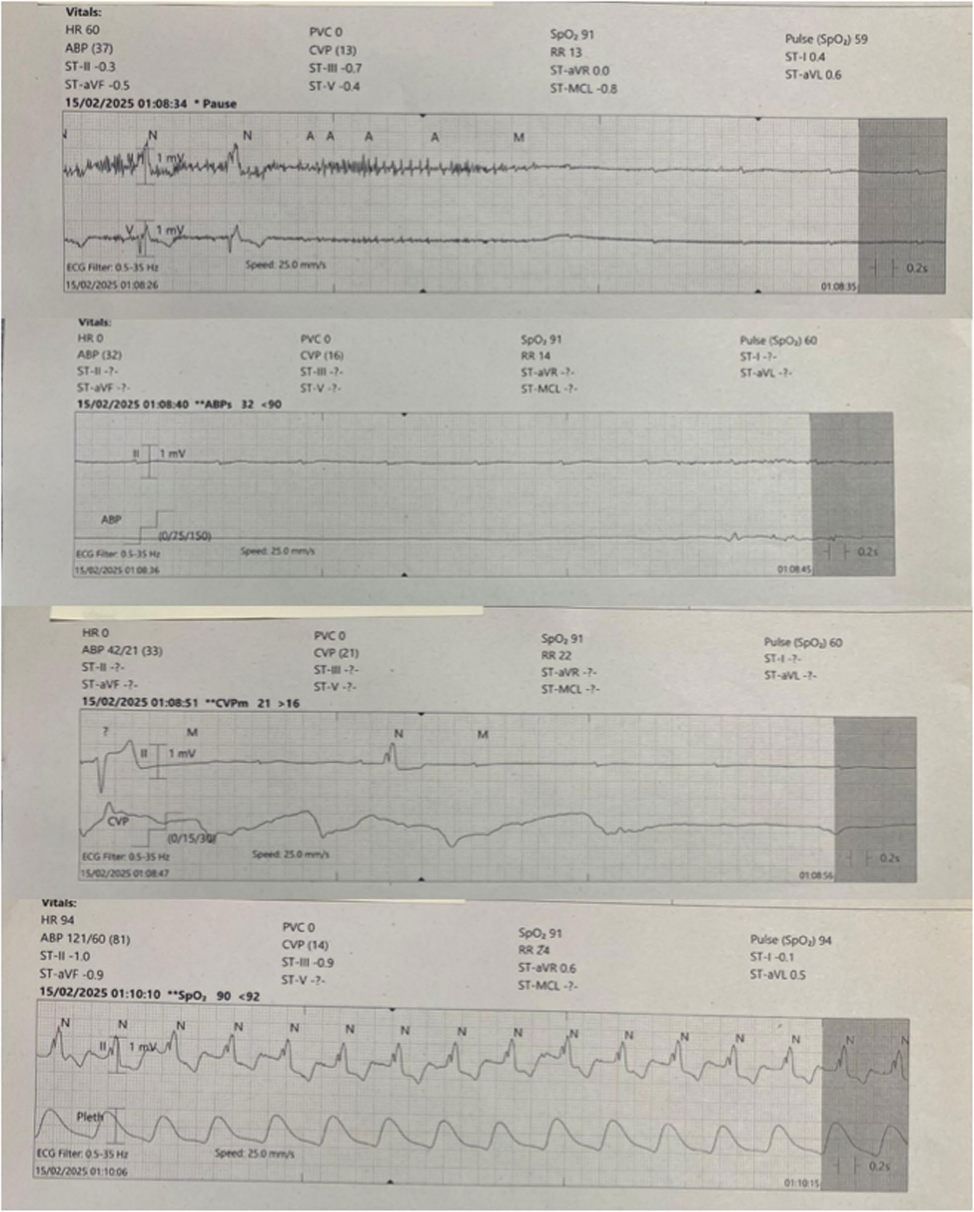
Chronological telemetry recordings demonstrating a period of sustained ventricular asystole followed by spontaneous return to sinus tachycardia

Ten hours following commencement of octreotide infusion, the patient sustained an approximately 30-second ventricular asystole with associated loss of cardiac output and resulting loss of consciousness. Two similar asystolic events were witnessed subsequently over the next 2 hours (30 and 10 seconds, respectively) (Fig. 1). The patient reported feeling generally unwell and nauseated following each return of spontaneous circulation. Examination following return of spontaneous circulation was unremarkable, and no dynamic changes were seen on 12-lead echocardiogram (ECG). Cardiology consult with review of telemetry recordings (as demonstrated in Fig. 1) concluded that the demonstrated rhythm likely demonstrated ventricular asystole or high-grade atrioventricular block. Sinus arrest was deemed unlikely given reliable presence of P-waves on telemetry recordings. Cardiology advised ongoing monitoring, monitoring of extended electrolytes, and consideration of isoprenaline infusion in the case of further episodes.

Octreotide infusion was stopped following suspicion of association owing to known side effects of sinus bradycardia, and previous published case reports of associated heart block. No other contributing medications were identified. The potential for direct endocardial stimulation from the tip of the central venous catheter was considered unlikely to be contributing to recurrent ventricular asystole owing to the catheter tip position being visible above the right atrium on chest X-ray. The patient was closely observed over the course of the subsequent 48 hours on ICU with no further episodes of ventricular asystole or loss of cardiac output following cessation of the octreotide infusion.

## Follow-up

The patient was reviewed by the inpatient cardiology team, and no routine inpatient or outpatient follow up was recommended. The patient has since been discharged from hospital, and has returned to their pre-admission functional status. They remain independent with all activities of daily living at 6 months following discharge from hospital.

## Discussion and conclusions

While generally well-tolerated, adverse cardiovascular effects with administration of intravenous octreotide have been reported previously. Little is understood regarding the mechanism contributing to octreotide-induced bradyarrhythmias. Somatostatin itself is proposed to have negative chronotropic effects on cardiac musculature, via reduction in intracellular cyclic AMP, reduced calcium uptake into the myocardium via inhibition of voltage-gated calcium channels, and reduced activation of potassium membrane conduction [7]. A small electrophysiological study involving 12 patients has demonstrated prolonging of sinoatrial node, intra-atrial, and atrioventricular conduction times, with associated slowing of the sinus cycle and lengthening effective refractory periods [8]. Animal studies have shown similar results; however, human trials have been extremely small and not generalizable [9, 10].

A previous history of cardiac surgery may also predispose to the arrhythmic side effects associated with intravenous octreotide. PAPVD repair is associated with arrhythmias in the early and late postoperative periods. These tend to be supraventricular tachyarrhythmias, and are most associated with surgical technique, with atrial arrhythmias and nodal rhythms occurring most commonly with larger incision into the cavo-atrial junction [11, 12]. Arrhythmias associated with administration of octreotide may be more common in patients with structural myocardial defects associated with previous cardiac surgery [11].

Previously published case reports have described occurrence of sinus bradycardias, various types of heart block and, rarely, asystole [13–15]. To our knowledge, this is the first case of octreotide-induced ventricular asystole reported in a postoperative patient with a previous history of congenital cardiac surgery. The case reports available, including ours, represent an exceedingly small proportion of anecdotal evidence without significant power and generalizability to form guidance on the requirement for enhanced monitoring in patient groups receiving intravenous octreotide.

In conclusion, the risk factors predisposing patients to octreotide-associated arrhythmias remain generally unknown. As a result, there is a paucity of guidance as to which patients require enhanced blood pressure and cardiac monitoring while receiving intravenous octreotide. Structural cardiac abnormalities such as those from prior cardiac surgery may increase susceptibility to conduction abnormalities in patients receiving intravenous octreotide. Monitoring of invasive blood pressure and telemetry may be warranted in these cases, allowing for early detection of arrhythmias and effects on cardiac output.

## Learning points

- Octreotide infusion can be associated with significant cardiac conduction abnormalities, including sinus bradycardia, heart block, and as described in this case, ventricular asystole.
- Structural cardiac abnormalities may increase risk of bradyarrhythmias and asystole with concurrent administration of intravenous octreotide.
- While there is a paucity of evidence regarding risk factors for cardiac arrhythmias associated with intravenous octreotide, enhanced cardiac and invasive blood pressure monitoring during octreotide infusion may be required in patients with previous history of cardiac surgery.

## Data Availability

Not applicable. This case report does not include any data.
